# The Nitrogen Cycle: A Large, Fast, and Mystifying Cycle

**DOI:** 10.1264/jsme2.ME3403rh

**Published:** 2019-09-25

**Authors:** Ken Takai

**Affiliations:** 1 Institute for Extra-cutting-edge Science and Technology Avant-garde Research (X-star), Japan Agency for Marine-Earth Science and Technology (JAMSTEC) 2–15 Natsushima-cho, Yokosuka 237–0061 Japan

If a story was written entitled the “Kingdom of Microbial Ecology”, how would the characters and plot be decided? Many microbiologists will agree with the following: the primary story setting involves a magnificent castle (a habitat) in fertile land with bright sunlight (the energy source), and the king and queen are the carbon and nitrogen cycles, respectively. Although this is an unusual way to begin a research highlight article in *Microbes and Environments*, the nitrogen cycle is one of the most important and commonly researched topics in the field of environmental microbiology.

Nitrogen is among 6 major essential elements of CHNOPS for life and composes the building blocks and intact molecules for metabolism (amino acids and proteins), heredity (nucleotides and nucleic acids), and other important biological functions. Among the essential elements, circulating volumes of biologically available nitrogen and phosphorus are slightly limited in many biospheres of the planet, and, thus, as a whole, the biological demand for nitrogen and phosphorus and their circulation speeds are markedly greater and faster than those for carbon (3). Furthermore, although the biological transformation pathways of nitrogen compounds are less diverse than those of carbon compounds when all organic forms are taken in consideration, the complexity of the catabolic and anabolic metabolism of inorganic nitrogen compounds and their synergetic processes are beyond those for carbon and cannot be compared to those of other biologically essential elements (9). This may, at least in part, explain the great interest in as well as difficulties associated with environmental microbiology research on the nitrogen cycle, which has encouraged and motivated many scientists to study it and resulted in a greater abundance of research articles and reviews on the nitrogen cycle and metabolism than on energy, carbon, and other elemental cycles and metabolism in the last decade (a quick Google scholar search shows 3,100,000 hits for “energy cycle/metabolism”, 2,830,000 hits for “carbon cycle/metabolism”, 2,470,000 hits for “nitrogen cycle/metabolism”, and 670,000 hits for “phosphorus cycle/metabolism”).

There are two large nitrogen pools on Earth, atmospheric molecular nitrogen (N_2_) and (biologically) reactive nitrogen (NO_3_, NH_4_, and organic nitrogens) (3). The biological nitrogen cycle mainly consists of internal interactions within the reactive nitrogen pool and in- and out-flow between reactive nitrogens and atmospheric N_2_ pools. Inter-connections between the two large nitrogen pools are primarily controlled by only two biological (microbial) processes, nitrogen fixation and denitrification (3), even though the impact of anthropogenic input into the terrestrial and marine reactive nitrogen pools on the global nitrogen cycle have recently increased due to fertilization and the burning of fossil fuels.

Major catabolic and anabolic microbial nitrogen metabolic pathways are shown in Fig. 1, the complexity of which has undoubtedly motivated scientific curiosity. In the past several years, many studies that have been published in *Microbes and Environments* have attempted to elucidate microbial nitrogen cycles at both the local and global scales of microbial habitats and communities.

Nitrogen fixation is a unique ability possessed by microorganisms called diazotrophs, and involves the conversion of very inert N_2_ to reactive nitrogens (including NH_3_) (2). The conversion of N_2_ to reactive nitrogens (such as NO) always occurs with lightning, which provides reactive nitrogens to the land and ocean (16). Japanese farmers previously noted a relationship between the annual abundance of lightning and rice crop yields, and referred to this lightning as “Inazuma” (the housewife for rice crop growth). However, this abiotic nitrogen fixation input has been estimated to account for only <1/10 of biological nitrogen fixation based on theoretical calculations and only approximately 10^−5^ based on laboratory experiments (3, 16). Thus, microbial nitrogen fixation and diazotrophs are key in the global nitrogen cycle and even on various scales in biological communities ever since the Earth became a planet of life approximately 4 Ga.

Nishihara and colleagues investigated nitrogen fixation in chemolithotrophic microbial communities in a hot spring stream in Japan (17–19). Chemolithotrophic nitrogen fixation at high temperatures (up to 92°C) has attracted scientists researching the early evolution of life and the nitrogen cycle, and deep-sea hyperthermophilic methanogens and their nitrogen fixation processes have been extensively examined (12, 20). The types of thermophilic diazotrophs supporting nitrogen sources for chemolithotrophic microbial communities in terrestrial geothermal environments lacking a significant reactive nitrogen input were previously unknown. A series of studies by Nishihara and colleagues revealed that nitrogen fixation occurred in thermophilic microbial mats along the hot spring stream at temperatures up to 75°C, and was not associated with the functions of thermophilic methanogenic and sulfate-reducing diazotrophs, previously known as thermophilic diazotrophs, based on activity measurements and molecular analyses (17, 19). Nishihara and colleagues also succeeded in isolating new thermophilic chemolithoautotrophs that potentially function as primary diazotrophs in microbial mat communities and showed that they were hydrogenotrophic and/or thiotrophic diazotrophs of the genus *Hydrogenobacter* (18). Genetic analyses of nitrogen fixation genes and phenotypes have also been performed on ecologically important diazotrophic microbes, such as a thermophilic cyanobacterium (26) and plant-associated actinobacterium (8). Using a metatranscriptomic approach, Masuda *et al.* (11) found that nitrogen fixation in paddy fields was primarily driven by deltaproteobacterial populations, such as *Anaeromyxobacter* and *Geobacter*, but not by other rhizospheric *Proteobacteria* and *Cyanobacteria* that were considered to be the major diazotrophs in paddy soils. Masuda *et al.*(11) identified true “Inazuma”, the housewife for rice crop growth, and this study was selected for the Most Valuable Paper (MVP) award of 2017 in *Microbes and Environments*. It is important to note that the diversity, abundance, and function of plant-associated diazotrophs are significantly controlled by the abundance and input of other reactive nitrogens (14).

In catabolic and anabolic metabolic pathways, the significance of intermediate nitrogen metabolites, such as nitrite (NO_2_), nitric oxide (NO) and nitrous oxide (N_2_O), has been recognized in many scientific and social contexts. Nitrification, denitrification, dissimilatory nitrate reduction to ammonium (DNRA), assimilatory ammonication, and anaerobic ammonium oxidation (Anammox) each through the transformation of these intermediate nitrogen metabolites and intermediates in natural microbial communities may be multi-directionally transformed by the complex nitrogen metabolism of various populations in response to intra- and extracellular physicochemical conditions and reaction dynamics through interspecies interactions (Fig. 1). Nakagawa *et al.*(15) reported the diversity and abundance of a nitrous oxide reductase gene (*nosZ*) in coastal eelgrass sediments and suggested that sulfur-oxidizing *Gammaproteobacteria* and *Bacteroidetes* populations contribute to N_2_O removal in eelgrass sediment microbiomes. Siqueira *et al.*(23) confirmed the previously proposed hypothesis that the different denitrification pathways and functions of similar *Bradyrhizobium* species in the soybean rhizosphere (*e.g*., with and without *nosZ*) respond to the physicochemical conditions of habitats (*e.g*., *in situ* O_2_ concentrations). Although nitrous oxide reduction (with and without *nosZ*) is not key for denitrification, the abundance of nitrate reduction (the expression of *napA*) may be more important. Jang *et al.*(5) also isolated the novel denitrifying bacterium *Bradyrhizobium nitroreducens* from rice paddy soil that hosted and co-expressed two types of nitrite reductase genes (*nirK* and *nirS*), and the majority of denitrifiers are known to possess and use each of the two types of nitrite reductases. These are also excellent examples of the complexity of the nitrogen cycle in natural microbial communities.

Besides denitrification, DNRA is considered to play an important role in energy metabolism with the nitrates and nitrites of microbial communities occurring at relatively electron-donor-enriched and/or electron-acceptor-limiting habitats based on the thermodynamic estimation of energy efficiency. Chutivisut *et al.* (1) showed that the denitrifying microbial community of activated sludge from a municipal wastewater treatment plant enriched DNRA populations when it was incubated under the condition of a high donor/acceptor ratio. Furthermore, *Microbes and Environments* recently published a number of studies on ecologically important nitrogen catabolism by Anammox and Anammox microbial communities (13, 22).

In many natural and anthropogenic habitats, nitrifying microbial communities are reliant on the close cooperation of two distinct groups, namely, that between ammonia- and nitrite-oxidizing metabolism (except for Comammox) (25) and also that between archaeal and bacterial populations. Ammonia-oxidizing archaea (AOA) play a significant role in natural environments and include phylogenetically and physiologically diverse members. Using a metagenomic analysis and the long-term enrichment of a thermophilic nitrifying microbial community obtained from a subsurface geothermal water stream, Kato *et al.* (6) demonstrated that previously uncultivated *Nitrocaldus* and *Aigarchaeota* members were enriched in the thermophilic ammonium-fed culture and may be involved in not only nitrification, but also denitrification in a long-term culture and indigenous habitats. Isoda *et al.* (4) and Nunoura *et al.* (21) reported the nitrifying archaeal phylotype diversity and potential niche separation of diverse *Thaumarchaeota* in Finnish and Japanese forest soils as well as in the world’s deepest Challenger Deep of the Mariana Trench. These AOA oxidize ammonia to hydroxylamine by ammonia monooxygenase (Amo); however, the enzymatic entity that catalyzes the oxidation of hydroxylamine to nitrite, corresponding to hydroxylamine dehydrogenase (Hao) in the case of ammonia-oxidizing bacteria (AOB), remains unclear (24). AOA are known to produce nitric oxide during their chemolithoautotrophic growth (10). Using the recombinant copper-containing nitrite reductase (NirK) of *Nitrososhaera viennensis* and enzymological characterization, Kobayashi *et al.* (7) confirmed that NirK is involved in the production of nitric oxide via hydroxylamine oxidation and nitrite reduction during chemolithoautotrophic growth. Under the experimental conditions described, no evidence of nitrite production from hydroxylamine by the recombinant NirK was obtained (7); however, this study provided key insights into the as-yet-unknown metabolic pathways and enzymatic entities of ammonia oxidation and nitric oxide production by AOA. This concise, but impacted study was selected for the MVP award of 2018 in *Microbes and Environments*.

The MVPs in *Microbes and Environments* in the last two years have been awarded to studies on the nitrogen cycle. Therefore, the large, fast, and complex nitrogen cycle has attracted the attention of many environmental microbiologists worldwide, and *Microbes and Environments* has significantly contributed to lifting the veil of secrecy surrounding “the Queen of the Kingdom of Microbial Ecology”.

## Figures and Tables

**Fig. 1:**
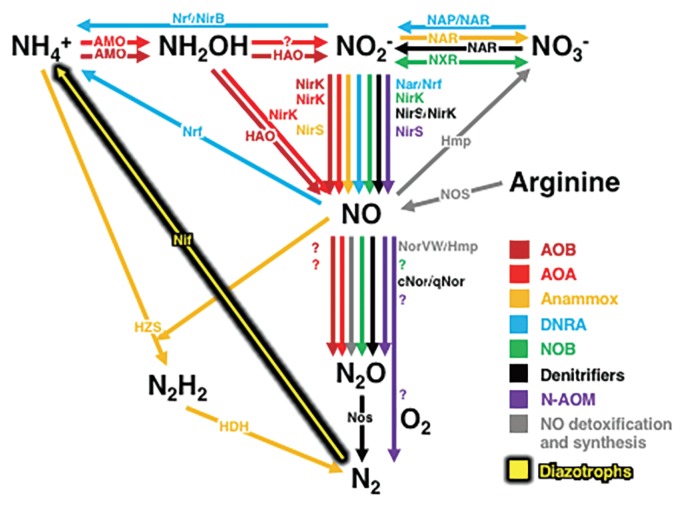
Microbial pathways in the nitrogen cycle. Abbreviations: AOB, ammonia-oxidizing bacteria; AOA, ammonia-oxidizing archaea; Anammox, anaerobic ammonium oxidation; DNRA, dissimilatory nitrate reduction to ammonium; NOB, nitrite-oxidizing bacteria; N-AOM, oxygenic nitrite-dependent anaerobic oxidation of methane. Key enzymes in these pathways are shown as follows: AMO, ammonia monooxygenase; HAO, hydroxylamine oxidoreductase; NirK, copper-containing nitrite reductase; NirS, cytochrome *cd*_1_ nitrite reductase; Nrf, cytochrome *c* nitrite reductase; NirB, cytoplasmic nitrite reductase; NAR, membrane-bound nitrate reductase; NAP, periplasmic nitrate reductase; NXR, oxidoreductase; NOS, nitric oxide synthase; Hmp, flavohemoglobins; NorVW, flavorubredoxin; cNor, nitric oxide reductase using c-type cytochromes as an electron donor; qNor, nitric oxide reductase using quinols as an electron donor; Nos, nitrous oxide reductase; HZS, hydrazine synthase; HDH, hydrazine dehydrogenase; Nif, nitrogenase. Unknown enzymatic entities and abiotic reaction steps are shown as question marks.
